# Nonlinear thermoelectric effects in high-field superconductor-ferromagnet tunnel junctions

**DOI:** 10.3762/bjnano.7.152

**Published:** 2016-11-03

**Authors:** Stefan Kolenda, Peter Machon, Detlef Beckmann, Wolfgang Belzig

**Affiliations:** 1 Karlsruher Institut für Technologie (KIT), Institut für Nanotechnologie, P.O. Box 3640, D-72021 Karlsruhe, Germany; 2 Department of Physics, University of Konstanz, D-78457 Konstanz, Germany

**Keywords:** spintronics, superconductor-ferromagnet hybrids, thermoelectricity

## Abstract

**Background:** Thermoelectric effects result from the coupling of charge and heat transport and can be used for thermometry, cooling and harvesting of thermal energy. The microscopic origin of thermoelectric effects is a broken electron–hole symmetry, which is usually quite small in metal structures. In addition, thermoelectric effects decrease towards low temperatures, which usually makes them vanishingly small in metal nanostructures in the sub-Kelvin regime.

**Results:** We report on a combined experimental and theoretical investigation of thermoelectric effects in superconductor/ferromagnet hybrid structures. We investigate the dependence of thermoelectric currents on the thermal excitation, as well as on the presence of a dc bias voltage across the junction.

**Conclusion:** Large thermoelectric effects are observed in superconductor/ferromagnet and superconductor/normal-metal hybrid structures. The spin-independent signals observed under finite voltage bias are shown to be reciprocal to the physics of superconductor/normal-metal microrefrigerators. The spin-dependent thermoelectric signals in the linear regime are due to the coupling of spin and heat transport, and can be used to design more efficient refrigerators.

## Introduction

Electrons in classical superconductors are bound in spin-singlet Cooper pairs, whereas ferromagnetic materials prefer parallel spin alignment. In nanoscale hybrid structures made of superconductors and ferromagnets, the competition of these antagonistic spin orders can be exploited to produce superconducting spintronics functionality [1–3]. Several promising spintronic effects have been theoretically predicted and subsequently experimentally observed. Examples are the odd-frequency triplet supercurrent [4–6] and fully spin-polarized quasiparticle currents [7–9]. Superconductor/normal-metal hybrid structures can also be used for local electron thermometry and microrefrigeration [10–11]. Recently, large spin-dependent thermoelectric effects were predicted [12–16] and experimentally observed [17] in superconductor/ferromagnet (SF) hybrid structures. These thermoelectric effects are linked to a coupling of spin and heat current, a phenomenon which has recently given rise to the field of spin caloritronics [18].

Previous work on thermoelectric effects in SF hybrids have concentrated on the regime of linear response of the electric and thermal currents to the difference in electric potential or temperature [12–14]. In that case the linear response coefficients – electrical and thermal conductance, Seebeck and Peltier coefficients – are related by the famous Onsager symmetry relations [12]. In particular these relate the Seebeck and Peltier coefficients to each other. In terms of practical applications the linear response coefficients are limited to devices with vanishing performance, due to the assumption of linearization in the thermodynamic forces. For example, the maximal possible Carnot efficiency |δ*T*|/*T* for a given temperature difference δ*T* at base temperature *T* is by definition much smaller than 1. Hence, a useful thermodynamic machine need to be run at finite power output, in which the linearization might not work anymore. A well-known application beyond the linear regime are normal-metal/insulator/superconductor (NIS) junctions under voltage bias close to the energy gap of the superconductor, which provide local electronic refrigeration [10–11]. Charge and spin transport in the nonlinear bias regime have also been investigated experimentally [8–9] and theoretically [19–23].

In this paper, we extend our previous theoretical [12–13] and experimental [17] work on thermoelectric effects in SF hybrid structures in a combined experimental and theoretical study of the nonlinear regime both as a function of thermal and voltage excitation. In particular, we elucidate the relation of thermoelectric currents to superconducting microrefrigerators by generalizing Onsager relations. Throughout this paper, we will use F, S, I and N to denote ferromagnetic, superconducting, insulating and normal-metal parts of our structures, e.g., FIS for a ferromagnet-insulator-superconductor junction.

## Theory

In the linear response regime the Seebeck and the Peltier coefficients are related by the Onsager reciprocity relation. Hence a measurement of one determines the other. This is not the case in the nonlinear regime anymore. In the following we derive a generalization of the Onsager relation in the nonlinear regime to evaluate the performance of mesoscopic cooling devices. Obviously this cannot be as general as the Onsager reciprocity, but relies on a concrete model of elastic transport. In the end it will be useful to evaluate the practically important heat current from the measure thermally induced charge current.

We consider a metal coupled to a superconductor by a tunnel contact, with normal-state tunnel conductance *G*_T_. The metal can be a normal metal or a ferromagnet, in which case the junction conductance has a finite spin polarization *P*. In that context the superconductor is kept at zero chemical potential, and both voltage bias *V* and thermal excitation δ*T* are applied to the normal-metal (or ferromagnet). All currents are counted flowing into the superconductor. We can in general express the charge and heat currents flowing out of the ferromagnet as

[1]
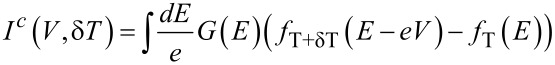


[2]
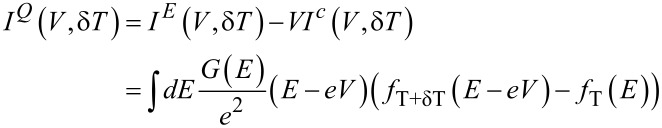


Here *G*(*E*) is the spectral conductance and *f_T_*(*E*) = (exp(*E*/*k*_B_*T*) + 1)^−1^ is the Fermi function at energy *E*. The spectral conductance is given by

[3]
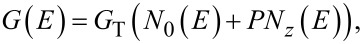


where *N*_0_ = (*N*_+_ + *N*_−_)/2, *N_z_* = (*N*_+_ − *N*_−_)/2, and the spin-resolved density of states in the superconductor is *N*_±_(*E*). We would like to point out that *N*_0_ is even in energy, while *N_z_* is odd in energy and gives rise to the broken particle–hole symmetry of *G*(*E*) for *P* ≠ 0. For the fits of the experimental data shown below, *N*_±_(*E*) is calculated from the standard model of high-field superconductivity [24] (see Supporting Information File 1 for details).

In the linear regime, i.e., for *V* → 0 and δ*T* → 0, Equation 1 can be written as

[4]
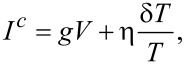


where *g* is the conductance, *T* is the average temperature, and η describes the thermoelectric current. η is related to the Seebeck coefficient *S* = −*V*/δ*T* measured in an open circuit by η = *SgT*. In general, however, *I^c^* is a nonlinear function of both δ*T* and *V*, and the thermoelectric transport coefficient can be generalized to

[5]
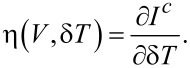


**Figure 1:**
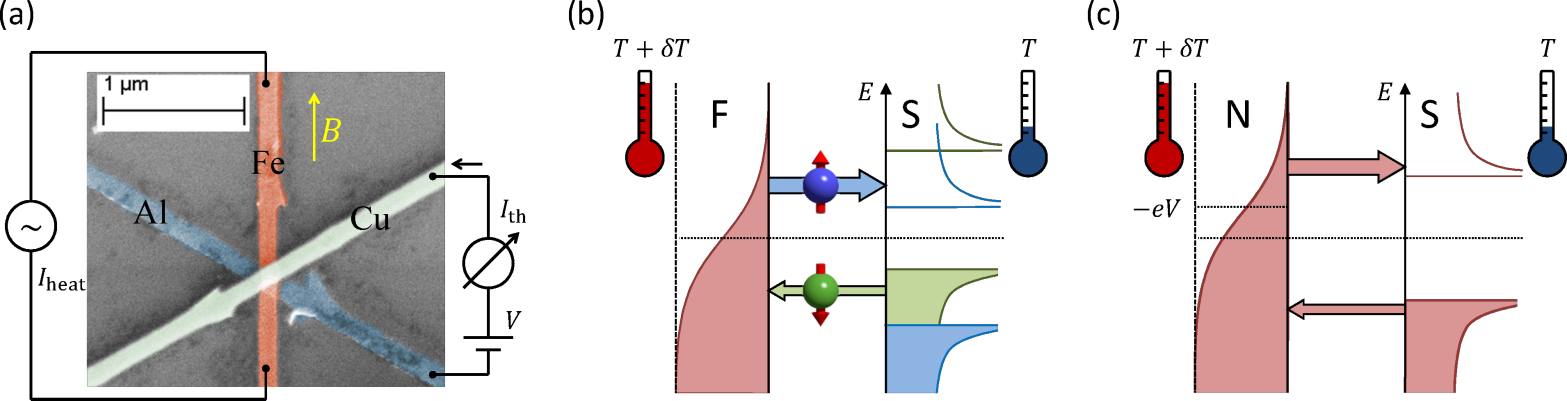
(a) False-color scanning electron microscopy image of one of our samples, together with the measurement scheme. The samples consist of a six-probe tunnel junction between a superconducting aluminum (Al) and a ferromagnetic (Fe) wire, with an overlaid copper (Cu) wire providing additional measurement leads. (b) Scheme of the generation of the linear thermoelectric effect in a FIS junction. (c) Scheme of the generation of the nonlinear thermoelectric effect in a NIS (or FIS) junction.

The physics of the thermoelectric current generation in a high-field FIS junction at *V* = 0 is shown schematically in Figure 1b. The Zeeman splitting of the quasiparticle states in the superconductor leads to a spin-dependent density of states (left). Heating of the ferromagnet leads to a flow of spin-up electrons at positive energy from occupied states in the ferromagnet into the superconductor, and a flow of spin-down electrons out of the superconductor into unoccupied states in the ferromagnet at negative energies (relative to the chemical potential of the superconductor). For finite spin polarization *P* of the junction conductance, the two currents are unequal, and therefore a net charge current flows across the junction, accompanied by both spin and heat currents. For *V* = 0, only the part of the spectral conductance *G*(*E*) which breaks particle–hole symmetry contributes to the thermoelectric current, i.e., the part proportional to *N_z_*(*E*).

At finite voltage bias *V*, schematically depicted in Figure 1c, the current through a NIS or FIS junction always depends on temperature, as the forward and backwards currents are always unequal. In this case the generalized nonlinear coefficient η also contains the temperature dependence of the regular voltage-driven tunnel current, and there is no simple relation to the symmetry of the spectral conductance. Nonlinear thermoelectric coefficients and their symmetries have also been discussed theoretically for superconductor/quantum dot systems [25–26].

One aim of this paper is to understand the relation of the experimentally observed η(*V*) to known results of thermal transport in superconductor hybrid structures. We therefore derive here a generalized relation between the charge current *I^c^* and the heat current *I^Q^* in the nonlinear regime. In the following derivation, we assume the spectral conductance to be independent of temperature and bias voltage. This is in general not always fulfilled, since, e.g., the superconducting gap Δ depends on temperature. However, this becomes mainly relevant close to *T_c_* and we will in the following neglect the temperature dependence. The following derivation will be based on the identity


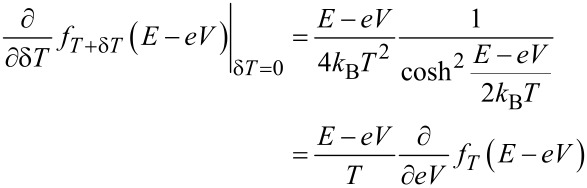


valid for arbitrary bias voltage. Hence we can write


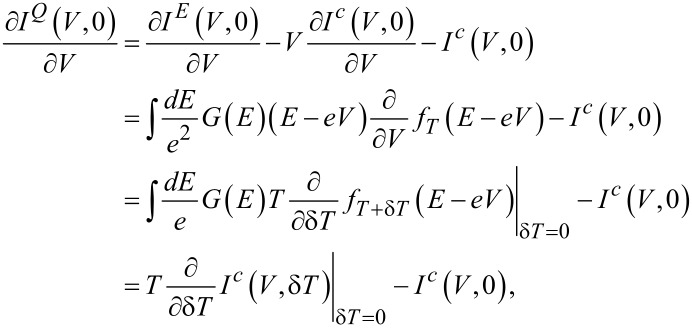


and finally

[6]
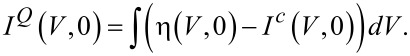


This is the main theoretical result and can directly be applied to the experimental data (see below).

## Experiment and Results

Our samples were fabricated by e-beam lithography and shadow evaporation. The central part is a tunnel junction between ferromagnetic iron and superconducting aluminum, with a thin aluminum oxide layer as tunnel barrier. An additional copper wire is overlaid to provide additional measurement leads, forming a six-probe junction. Figure 1a shows a false-color scanning electron microscopy image of one of our samples, together with the measurement scheme. The wire widths are around 200 nm, and the film thicknesses are *t*_Al_ ≈ 20 nm, *t*_Fe_ ≈ 15–20 nm and *t*_Cu_ ≈ 50 nm for the aluminum, iron and copper wires, respectively.

Transport measurements were carried out in a dilution refrigerator at temperatures down to 50 mK, with an applied in-plane magnetic field *B* parallel to the iron wire. To create a temperature difference δ*T* across the junction, we pass a heater current *I*_heat_ along the ferromagnetic wire. The local temperature of the ferromagnet at the junction can be described by [10]

[7]
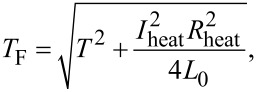


where *T* is the electronic base temperature without heating, *R*_heat_ is the resistance of the ferromagnetic wire, and *L*_0_ = π^2^*k*_B_^2^/3*e*^2^ is the Lorenz number. We calibrate the dependence of *T*_F_ on *I*_heat_ by measuring the differential conductance of the junction while applying a dc heater current. The actual temperature difference δ*T* is usually slightly smaller than δ*T*_F_ = *T*_F_ − *T* obtained from the calibration measurements due to indirect heating of the superconductor. We typically find δ*T* ≈ 0.8δ*T*_F_. Details of the temperature calibration can be found in [17] and Supporting Information File 1. To measure the thermoelectric current through the junction, we apply a low-frequency ac heater current. Since the heating power is proportional to *I*^2^, this generates a thermal excitation on the second harmonic of the excitation frequency. We monitor the second harmonic of the current *I*_th_ through the junction, from which we can obtain a finite-difference approximation η = *TI*_th_/δ*T* of the differential nonlinear coefficient η given by Equation 5.

In our previous work [17], we focused on the measurement of η for a fixed thermal excitation δ*T* at *V* = 0. Here, we elucidate the nonlinear regime both as a function of thermal excitation and voltage bias. We show data from three samples, two with ferromagnetic junctions (FIS1 and FIS2), and a reference sample where the iron wire is replaced by copper to form a nonmagnetic junction (NIS). Details of the sample parameters and characterization can be found in [17].

**Figure 2:**
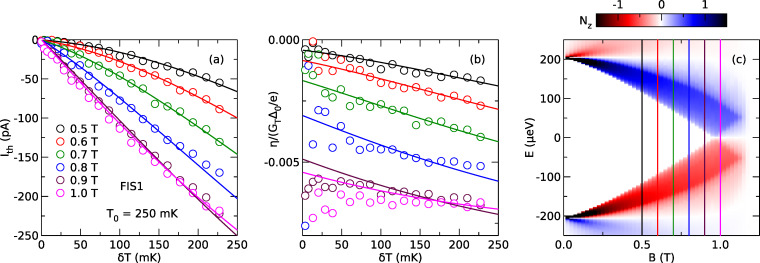
(a) Thermoelectric current *I*_th_ as a function of thermal excitation amplitude δ*T* for different magnetic fields *B* (sample FIS1). Lines are fits of Equation 1 to the data. (b) Thermoelectric transport coefficient η normalized to *G*_T_Δ_0_/*e* corresponding to the data in panel (a) Lines are the same fits as in (a). (c) *N_z_* = (*N*_↑_ − *N*_↓_)/2 as a function of applied field *B* and energy *E*. Vertical lines indicate the applied fields *B* for the data in panels (a) and (b).

First, we would like to focus on the dependence on thermal excitation. Here, we probe the nonlinearity by changing the excitation amplitude δ*T*. This is of interest for thermometry applications, where one would like to have a large, and preferably linear, response to a small but finite temperature difference. In Figure 2a, we show the thermoelectric current *I*_th_ as a function of δ*T* for different magnetic fields *B* at a base temperature *T*_0_ = 250 mK measured in sample FIS1, together with fits to Equation 1 (details of the data analysis can be found in Supporting Information File 1). At small fields, the thermoelectric current has a nonlinear dependence on the excitation. This can be understood by considering Figure 2c, where *N_z_* is plotted as a function of magnetic field and quasiparticle energy (using parameters derived from conductance experiments on the sample, see Supporting Information File 1). At small fields, the superconductor has an energy gap, and only the high-energy tail of the Fermi distribution contributes to the thermoelectric current. Due to the exponential energy dependence of the Fermi distribution, the current shows a nonlinear increase with increasing thermal excitation. Upon increasing the field, the gap decreases, and *I*_th_ consequently increases. At about *B* = 1 T, the gap vanishes, and *I*_th_ becomes largest and is now an almost linear function of δ*T*. In Figure 2b, we show the corresponding thermoelectric coefficient η = *TI*_th_/δ*T*, normalized to *G*_T_Δ_0_/*e*, where *G*_T_ = 275 μS is the normal-state junction conductance, and Δ_0_ = 208 μeV is the pair potential of the superconductor at *T* = 0 and *B* = 0. η has a weak dependence on the excitation δ*T* at small fields, and is nearly constant at high fields, reflecting the linearity of *I*_th_(δ*T*) at high fields.

**Figure 3:**
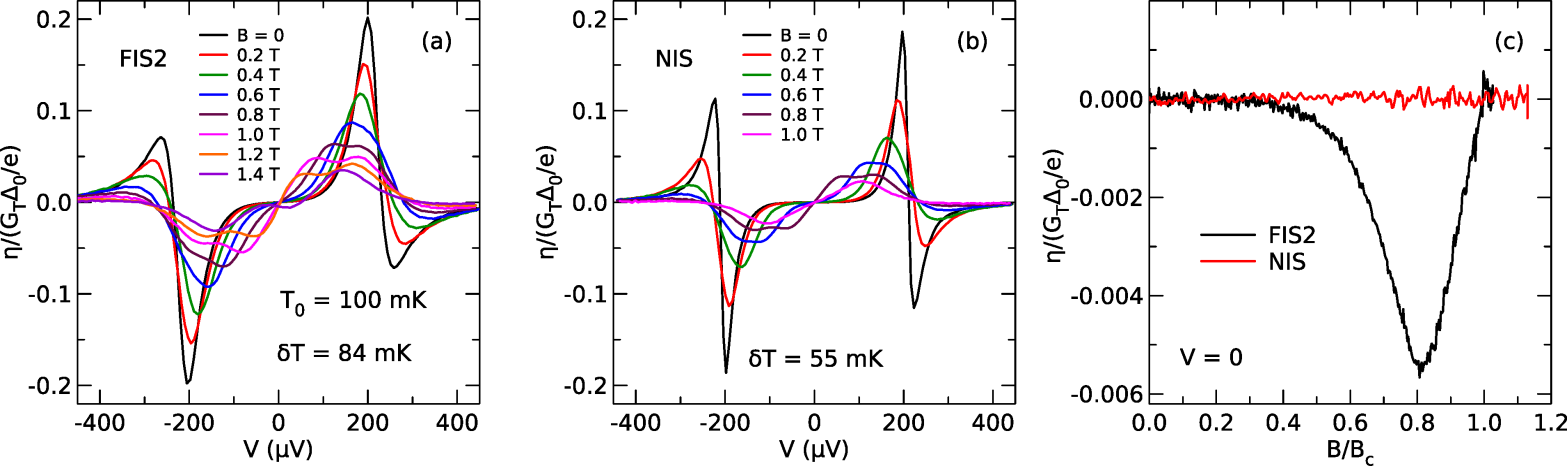
Thermoelectric transport coefficient η normalized to *G*_T_Δ_0_/*e* as a function of bias voltage *V* for different applied magnetic field *B*. (a) Data for a ferromagnetic junction (sample FIS2). (b) Data for a nonmagnetic junction (sample NIS). (c) Data at *V* = 0 for both samples as a function of normalized applied field *B*/*B*_c_.

Now, we focus on the dependence on bias voltage *V*. In Figure 3, we compare the nonlinear thermoelectric coefficient η as a function of voltage bias *V* for two samples, one with a ferromagnetic junction (a), and one with a normal–metal junction (b). η is plotted for fixed thermal excitation δ*T* at different magnetic fields. In Figure 3c, we show η(*V* = 0) as a function of field for comparison. While the nonmagnetic sample does not show a linear thermoelectric effect (due to the particle–hole symmetry of the spectral conductance of a NIS junction), both samples show a large nonlinear effect, even at zero applied field. Note that the overall signal scale in panels (a) and (b) is about two orders of magnitude larger than in panel (c), and that the linear thermoelectric effect at *V* = 0 is hardly visible on the scale of panels (a) and (b). The nonlinear coefficient of the NIS sample is an odd function of bias (only the *N*_0_ term in *G*(*E*) contributes). The data for the FIS sample have no simple symmetry (both *N*_0_ and *PN_z_* contribute), but are still dominated by the odd contribution due to the small *P* in our junctions. To understand the physical meaning of the nonlinear coefficient, we now relate it to the heat current using Equation 6.

**Figure 4:**
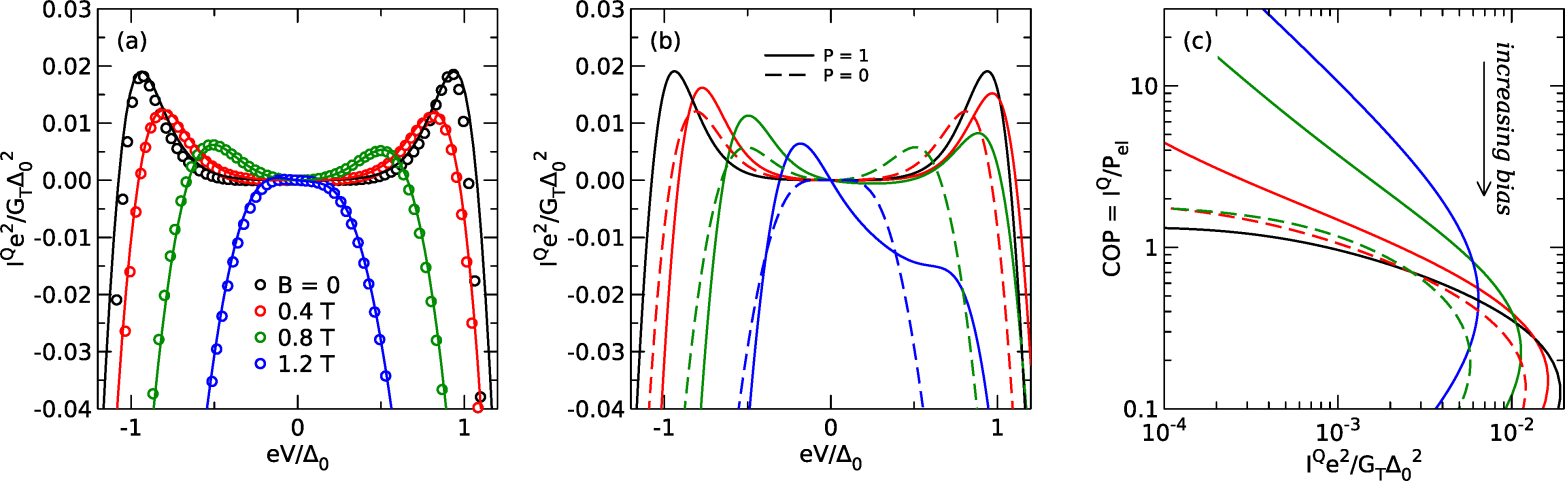
(a) Normalized cooling power *I^Q^e*^2^/*G*_T_Δ_0_^2^ as a function of normalized bias voltage *eV*/Δ_0_ for different magnetic fields *B*. (b) Predicted cooling power for the same device assuming *P* = 0 (NIS cooler) and *P* = 1 (ideal FIS Peltier cooler) as a function of normalized bias voltage. (c) Predicted coefficient of performance as a function of normalized cooling power for the same parameters as panel (b) and *V* < 0.

In Figure 4a, we show the cooling power *I^Q^* predicted from the measured thermoelectric coefficient η and dc current *I^c^* of sample FIS2 using Equation 6. Symbols are the results of the analysis of the experimental data, while lines are fits using Equation 2 directly. All fits are calculated at *T* = 250 mK using self-consistent parameters (see Supporting Information File 1 for details). The data and fits are in good agreement, showing that the cooling power can be inferred from the measured thermoelectric coefficient in the nonlinear regime. We would like to point out that the analysis is valid strictly speaking only for δ*T* → 0, whereas the experiment is necessarily carried out at finite δ*T*. However, as can be seen in Figure 2b, η depends only weakly on δ*T* for the experimental conditions, and we therefore neglect this dependence here. Also, the analysis yields the predicted cooling power for δ*T* = 0, and the actual cooling power under finite δ*T* will be smaller due to the backflow of heat via the thermal conductance of the junction. At *B* = 0, without spin splitting and consequently without linear thermoelectric effect, the predicted cooling power has the typical bias dependence of NIS microrefrigerators [10], with maximum cooling power for *eV* ≈ Δ. Upon increasing the field, the maximum of the cooling power shifts to smaller bias and decreases. Note that the Peltier cooling at zero bias due to the linear thermoelectric effect is too small to be resolved in this plot due to the low spin polarization *P* = 0.08 of our junction. Using the sample parameters of the fits shown in Figure 4a, we can now compare the predicted cooling power of a NIS cooler and an idealized FIS cooler with *P* = 1 in Figure 4b. As can be seen, there is no difference between NIS and FIS at *B* = 0. At finite field, the FIS cooler exhibits a linear Peltier contribution to the cooling power, which is largest at *B* = 1.2 T, roughly where the gap in the excitation spectrum of the superconductor vanishes. Under these conditions, the FIS Peltier cooler outperforms the NIS cooler at small bias. It is convenient to define the coefficient of performance *COP* for a cooler as the ratio *COP* = *I^Q^*/*P*_el_ = *I^Q^*/*I^c^V* of the cooling power and the electric input power of the device [27]. To make the improved performance of the FIS cooler more clear, we also plot the coefficient of performance as a function of cooling power in Figure 4c. The FIS cooler has superior efficiency over a wide range of cooling powers.

## Discussion

The thermoelectric current is largest and has a linear dependence on excitation at the magnetic field where the spectral gap of the superconductor vanishes. These conditions are therefore potentially useful for applications in thermometry or cooling. One possible way to improve performance is therefore to increase the spin splitting of the density of states by spin-active scattering with a ferromagnetic insulator [28–29], which is known to enhance nonequilibrium spin transport in nanoscale superconductors [30]. Also, performance can be improved by using ferromagnetic insulators as spin-filter tunnel junctions, with a degree of spin polarization *P* ≈ 100% [31–32].

At finite voltage bias, we find large thermoelectric signals for both FIS and NIS structures. Our analysis based on a generalized reciprocity relation shows that the generation of the thermoelectric signal is directly related to the cooling power of NIS microrefrigerators [10–11]. Further theoretical modeling shows that for an idealized FIS cooler with *P* = 100%, the thermodynamic efficiency can be greatly improved over NIS coolers. Future devices may include local control of the spin-splitting using the proximity effect with ferromagnetic insulators [30,33], or new thermoelectric multi-terminal devices [12–13].

## Supporting Information

File 1 Details of experimental procedures and theoretical model.
