# Supplementary material for: Nonlinear thermoelectric effects in high-field superconductor-ferromagnet tunnel junctions
Source: Beilstein J Nanotechnol. 2016 Nov 3;7:1579–85. doi: 10.3762/bjnano.7.152 (PMC5238697; doi:10.3762/bjnano.7.152)
Supplement: File 1 — Details of experimental procedures and theoretical model. [file Beilstein_J_Nanotechnol-07-1579-s001.pdf]

Supporting Information

for

Nonlinear thermoelectric effects in high-field superconductor-ferromagnet tunnel junctions

Stefan Kolenda[1], Peter Machon[2], Detlef Beckmann*[1] and Wolfgang Belzig*[2]

Address: [1]Karlsruher Institut für Technologie (KIT), Institut für Nanotechnologie, P.O. Box 3640, D-72021 Karlsruhe, Germany and [2]Department of Physics, University of Konstanz, D-78457 Konstanz, Germany

Email: Detlef Beckmann - detlef.beckmann@kit.edu, Wolfgang Belzig - wolfgang.belzig@uni-konstanz.de

* Corresponding author

Details of experimental procedures and theoretical model

Theoretical model

The spectral properties of a superconductor subjected to a high magnetic field can be determined by solving the implicit equation [1-3]

$$E+i\Gamma\pm h=\Delta u_{\pm}-\alpha_{\mathrm{orb}}\frac{u_{\pm}}{\sqrt{1-u_{\pm}^{2}}}+\alpha_{\mathrm{so}}\frac{u_{\pm}-u_{\mp}}{\sqrt{1-u_{\mp}^{2}}}. \tag{S1}$$

for the complex quantities $u_{\pm}$, where E is the energy, Γ is a phenomenological life-time broadening parameter (Dynes parameter [4]), h is the spin splitting, Δ is the pair potential, α_{orb} is the orbital pair-breaking parameter, and $\alpha_{\mathrm{so}}=\hbar/3\tau_{\mathrm{so}}$ is the spin-orbit scattering rate. For a thin film in a parallel magnetic field B, orbital pair-breaking is given by [5]

$$\alpha_{\mathrm{orb}}=\frac{\Delta_0}{2}\left(\frac{B}{B_{\mathrm{c,orb}}}\right)^{2}, \tag{S2}$$

where $\Delta_0=\Delta(B=0,T=0)$ is the pair potential at zero temperature and zero field, and $B_{\mathrm{c,orb}}$ is the critical field that would be observed at $T=0$ in the absence of spin splitting (the actual critical field is smaller due to the pair-breaking effect of the spin splitting h). The spin-resolved density of states is given by

$$N_{\pm}(E)=\mathrm{Re}\left(\frac{u_{\pm}}{\sqrt{u_{\pm}^{2}-1}}\right). \tag{S3}$$

For a superconductor-ferromagnet junction with normal-state tunnel conductance $G_{\mathrm{T}}=G_{+}+G_{-}$, the spectral conductance $G(E)$ in the superconducting state is given by

$$G(E)=G_{+}N_{+}(E)+G_{-}N_{-}(E)=G_{\mathrm{T}}\left(N_0(E)+PN_z(E)\right), \tag{S4}$$

where $N_0=(N_{+}+N_{-})/2$, $N_z=(N_{+}-N_{-})/2$, and $P=(G_{+}-G_{-})/(G_{+}+G_{-})$. For the self-consistent calculation of Δ and h, we use the model of Alexander et al. [3], which includes the

effect of Fermi-liquid renormalization on the spin splitting h. In the normal state, $h = \mu_B B/(1+G_0)$ with $G_0 = 0.3$ for aluminum [3,6], i.e., the spin splitting is reduced by interaction.

Experimental procedures and fits

To obtain the spectral properties of the superconductor, we measured the differential conductance dI/dV at different temperatures and magnetic fields using the standard ac technique, with a voltage excitation of a few μV at frequency $f \approx 138$ Hz. Examples of the spectra and details of the fits can be found in [7]. Fitting the conductance spectra yields all parameters for the spectral properties. In Figure S1 we show explicitly the model results for the fit parameters used in the main text. Figure S1(a) and (b) show the self-consist Δ and h as a function of applied field B corresponding to the plots in Figure 2 and Figure 4 of the main text. Figure S1(c) shows the density of states for the plots in Figure 4 of the main text.

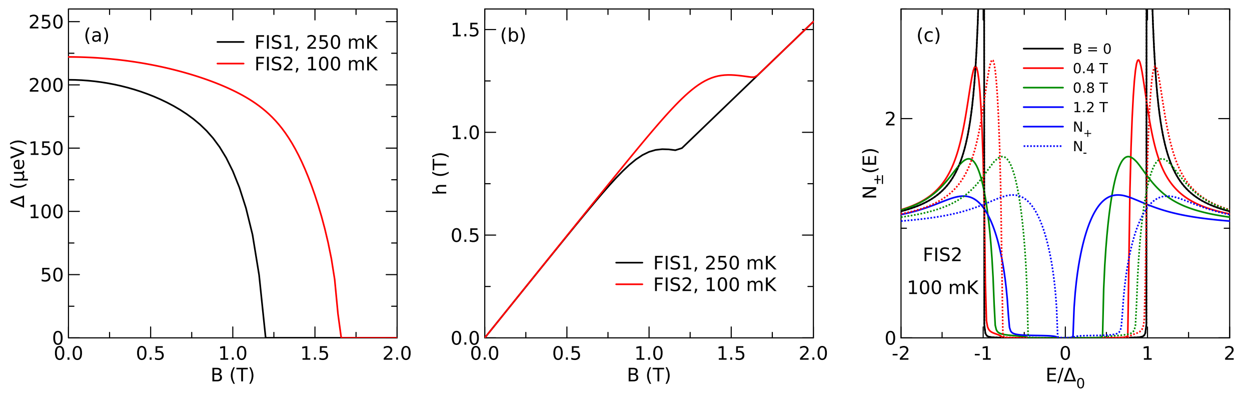

Figure S1: (a) self-consistent pair potential Δ and (b) self-consistent spin splitting h as a function of the applied field B. Data correspond to sample FIS1 at $T_0 = 250$ mK (Figure 2 of the main text) and sample FIS2 at $T_0 = 100$ mK (Figure 4 of the main text). (c) spin-resolved density of states $N_\pm(E)$ for sample FIS2 at $T_0 = 100$ mK for different magnetic fields B, corresponding to Figure 4 of the main text. Solid and dotted lines are N_+ and N_-, respectively.

Heater calibration was performed by measuring dI/dV with an additional dc heater current I_{heat} applied to the sample. The temperature T_F of the ferromagnet was then obtained by fitting the thermal smearing of dI/dV, keeping all other parameters fixed. Calibration curves at different bath temperatures T_0 for sample FIS1 are shown in Figure S2(a). The electronic temperature in the

absence of (deliberate) heating is slightly enhanced over the bath temperature T_0, especially at low temperatures. This can be attributed to heating due to incomplete filtering of the measurement lines. The lines are fits to eq. (7) of the main text, with the electronic base temperature T and the effective resistance R_{heat} of the heater wire as fit parameters.

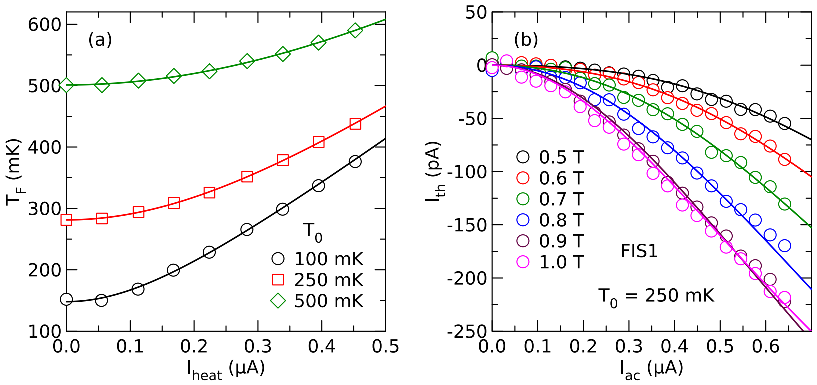

Figure S2: (a) Temperature T_F of the ferromagnet as a function of dc heater current I_{heat} for sample FIS1 at different base temperatures T_0. (b) Thermoelectric current I_{th} as a function of ac heater excitation I_{ac} (raw data of Figure 2 of the main text).

The thermoelectric experiments were carried out with an ac heater current $I_{\mathrm{heat}} = I_{\mathrm{ac}} \sin(2\pi f t)$ at frequency $f \approx 138$ Hz and amplitudes up to about 0.7 μA. Since the heater power is proportional to I_{heat}^2, the thermoelectric current I_{th} was measured at frequency $2f$. We checked that the thermoelectric signal was independent of excitation frequency for 38 Hz $\lesssim f \lesssim$ 250 Hz. According to the temperature calibration fits, the peak-to-peak thermal excitation is then given by

$$\delta T_F = \sqrt{T^2 + \frac{I_{\mathrm{ac}}^2 R_{\mathrm{heat}}^2}{4L_0}} - T. \tag{S5}$$

With the known spectral properties, junction characteristics and heater calibration, we can in principle calculate the expected thermoelectric effect without free parameters. This parameter-free calculation overestimates the measured effect by about 10-20%. This can be attributed to the fact that the temperature T_S of the superconductor is also increased indirectly by the heat current driven by δT_F (See [7] for a detailed discussion and experimental estimate of T_S). Therefore, the actual thermal excitation δT is slightly smaller than the one obtained from the calibration. Figure S2(b) shows the

raw data I_{th} for Figure 2 of the main text as a function of heater excitation I_{ac}. The lines are fits to eq. (1) of the main text, where we have accounted for the reduced δT by setting $\delta T = \alpha \delta T_F$ with α as the only fit parameter ($\alpha = 0.83$ for all fits). The coefficient η is then calculated as $\eta = I_{th}\overline{T}/\delta T$ with the average temperature $\overline{T} = T + \delta T/2$.

References

1. Maki, K. *Prog. Theor. Phys.* **1964**, *32* (1), 29–36. doi:10.1143/PTP.32.29.

2. Meservey, R.; Tedrow, P. M.; Bruno, R. C. *Phys. Rev. B* **1975**, *11* (11), 4224–4235. doi:10.1103/PhysRevB.11.4224.

3. Alexander, J. A. X.; Orlando, T. P.; Rainer, D.; Tedrow, P. M. *Phys. Rev. B* **1985**, *31* (9), 5811–5825. doi:10.1103/PhysRevB.31.5811.

4. Dynes, R. C.; Narayanamurti, V.; Garno, J. P. *Phys. Rev. Lett.* **1978**, *41* (21), 1509–1512. doi:10.1103/PhysRevLett.41.1509.

5. Maki, K. *Prog. Theor. Phys.* **1964**, *31* (5), 731–741. doi:10.1143/PTP.31.731.

6. Tedrow, P. M.; Kucera, J. T.; Rainer, D.; Orlando, T. P. *Phys. Rev. Lett.* **1984**, *52* (18), 1637–1640. doi:10.1103/PhysRevLett.52.1637.

7. Kolenda, S.; Wolf, J., M. Beckmann, D. *Phys. Rev. Lett.* **2016**, *116* (9), 097001. doi:10.1103/PhysRevLett.116.097001.
